# Knockdown of YAP/TAZ Inhibits the Migration and Invasion of Fibroblast Synovial Cells in Rheumatoid Arthritis by Regulating Autophagy

**DOI:** 10.1155/2020/9510594

**Published:** 2020-10-22

**Authors:** Wei Zhou, Qin Shen, Hui Wang, Jie Yang, Chen Zhang, Zijing Deng, Keyan Wu, Yang Zhou, Jing Zeng, Yu Zhang, Weigan Shen

**Affiliations:** ^1^Department of Cell Biology, School of Medicine of Yangzhou University, Yangzhou, China; ^2^Department of Rheumatology, Affiliated Hospital of Yangzhou University, Yangzhou, China; ^3^Department of Rheumatology, The Second People's Hospital of Lianyungang, Lianyungang, China; ^4^Jiangsu Co-Innovation Center for Prevention and Control of Important Animal Infectious Diseases and Zoonoses, Yangzhou University, Yangzhou, China; ^5^Jiangsu Key Laboratory of Integrated Traditional Chinese and Western Medicine for Prevention and Treatment of Senile Diseases, Yangzhou University, Yangzhou, China

## Abstract

The purpose of this study was to investigate the effect of knockdown of the yes-associated protein (YAP) and transcriptional coactivator with PDZ-binding motif (TAZ) on the migration and invasion of the rheumatoid arthritis fibroblast-like synoviocytes (RA-FLS) and to preliminarily elucidate the mechanisms between YAP/TAZ and autophagy in the migration and invasion of RA-FLS. RA-FLS stable knockdown of YAP or TAZ was successfully established by using lentiviral-mediated gene knockdown techniques. Wound healing assay and Transwell assay were used to evaluate the effect of knockdown of YAP or TAZ on the migration and invasion of RA-FLS. Reverse transcription quantitative real-time polymerase chain reaction (RT-qPCR) and western blotting assays were performed to examine the expression of indicated genes. The results showed that YAP and TAZ were upregulated in RA-FLS, and knockdown of YAP or TAZ inhibited the migration and invasion, reduced the expression of N-cadherin and Vimentin, and increased the accumulation of E-cadherin and *β*-catenin in RA-FLS. Our results also demonstrated that knockdown of YAP or TAZ promoted autophagy which increased the accumulation of LC3B-II and ULK1 and decreased the amount of SQSTM1/p62 in RA-FLS. Furthermore, our data displayed that inhibition of autophagy either with 3-MA or CQ can partially reverse the decrease of migration and invasion induced by YAP and TAZ knockdown in RA-FLS. Our experiments preliminarily revealed that YAP/TAZ and autophagy play important roles in the migration and invasion of RA-FLS, which might provide novel targets for the treatment of RA.

## 1. Introduction

Rheumatoid arthritis (RA) is a chronic, systemic autoimmune disease characterized by inflammation of the synovium, leading to destruction of cartilage and underlying bone. Evidence is accumulating that the fibroblast-like synoviocytes from RA patients (RA-FLS) play a pivotal role in the pathogenesis of RA [1]. RA-FLS exhibit some tumor cell-like characteristics, such as excessive proliferation, migration, and invasion; anchorage-independent proliferation; and lack of contact inhibition in vitro [2–4], and can migrate and invade the articular cartilage and bone [5], contributing to the destruction of cartilage and bone and joint damage through secretion of proinflammation cytokines and production of the proteolytic enzymes such as matrix metalloproteinases (MMPs) [6, 7]. Therefore, understanding and investigating the factors that regulate the migration and invasion of RA-FLS might provide novel targets for RA therapy.

The Hippo signaling, originally identified as an evolutionarily conserved signaling pathway, is known to be involved in tissue development, growth, and organ size control by regulating cell proliferation and survival [8, 9]. YAP (yes-associated protein) and TAZ (transcriptional coactivator with PDZ-binding motif) are downstream effectors of the Hippo signaling pathway [10, 11]. Both YAP and TAZ are transcriptional coactivators and can translocate into the nucleus and interact with transcription factors including TEA domain family members (TEADs) to regulate the expression of related genes that participate in the regulation of cell proliferation, epithelial-mesenchymal transition (EMT), cell motility, and cell fate [12, 13].

Autophagy is a highly conserved intracellular lysosome-dependent catabolic degradation pathway characterized by the isolation of cytoplasmic unnecessary or dysfunctional components in the autophagosome and subsequent fusion of the autophagosome with the lysosome and plays crucial roles in maintaining cellular homeostasis [14]. Emerging evidences indicate that autophagy plays a crucial role in cell migration and EMT [15, 16] and seems to be involved in the pathogenesis of several autoimmune diseases, including rheumatoid arthritis (RA) [17, 18]; however, the specific mechanism of autophagy in the pathogenesis of RA remains to be explored [19, 20]. Since YAP/TAZ has been shown to play an important role in cell migration, invasion, and EMT in a variety of human cancers and immune system diseases [21, 22] and dysregulation of autophagy in RA-FLS has also been demonstrated [23, 24], the functional interaction between autophagy and YAP/TAZ-mediated migration and invasion in RA-FLS remains to be further analyzed.

In the present study, we investigated the functional connection between the autophagy and cell motility induced by YAP knockdown and TAZ knockdown in RA-FLS by using lentiviral-mediated gene knockdown techniques. Here, we confirmed that either YAP or TAZ knockdown promoted autophagy and reduced migratory and invasive ability of RA-FLS. Most importantly, we also found that the loss of YAP or TAZ inhibited cell motility by activating autophagy in RA-FLS.

## 2. Materials and Methods

### 2.1. Cell Lines and Culture Conditions

Human RA-FLS was purchased from Shanghai Saiqi Biotech Company (Shanghai, China), normal FLS (NC-FLS) was purchased from Guangzhou Jennio Biotech Company (Guangzhou, China), and human embryonic kidney cell line 293T (HEK293T) was obtained from the Institute of Biochemistry and Cell Biology, Chinese Academy of Sciences (Shanghai, China). Cells were maintained in Dulbecco's modified Eagle's medium (DMEM, HyClone, Beijing, China) containing 12% fetal bovine serum (FBS, Gibco, Carlsbad, CA, USA) and 1% penicillin-streptomycin in an incubator at 37°C with 5% CO_2_.

### 2.2. Plasmid Construction, Lentivirus Production, and Infection

We followed the methods of Zhou et al. to generate the lentiviruses encoding YAP- and TAZ-specific shRNA and control shRNA, respectively [25]. Briefly, YAP- and TAZ-specific or control shRNA sequences were designed as follows: shYAP: sense, 5′-GATCCGCTGGTCAGAGATACTTCTTAATTCAAGAGATTAAGAAGTATCTCTGACCAGCTTTTTTA-3′ and antisense, 5′-CGCGTAAAAAAGCTGGTCAGAGATACTTCTTAATCTCTTGAATTAAGAAGTATCTCTGACCAGCG-3′; shTAZ: sense, 5′-GATCCGCGATGAATCAGCCTCTGAATTTCAA GAGAATTCAGAGGCTGATTCATCGCTTTTTTA-3′ and antisense, 5′-CGCGTAAAAAA GCGATGAATCAGCCTCTGAATTCTCTTGAAATTCAGAGGCTGATTCATCGCG-3′; and shC: sense, 5′-GATCCTGGTTTACATGTCGACTAATTCAAGAGATTAGTCGACATGT AAACCATTTTTTA-3′ and antisense, 5′-CGCGTAAAAAATGGTTTACATGTCGACTA ATCTCTTGAATTAGTCGACATGTAAACCACG-3′. The oligonucleotides were synthesized and annealed and then were inserted into the *Bam*H I and *Mlu* I sites of the pLent-U6-Puro empty vector (Vigene Biosciences Inc., Rockville, MD, USA). Lentiviral particles were packaged in HEK293T cells by transfection with the control shRNA vectors, shRNA targeting YAP, or TAZ constructs together with the lentiviral packaging vector pSPAX2 and pMD2G (Addgene Inc.) using EndoFectin Lenti reagent (GeneCopoeia, USA). The supernatant containing the lentiviral particles was harvested, concentrated, and used to infect cells. For infection of RA-FLS, cells were transduced with indicated lentiviruses in the presence of 8 *μ*g/ml of polybrene for 48 h and then selected with 2 *μ*g/ml of puromycin (Sigma-Aldrich Co., St. Louis, MO, USA) for 14 days to generate stable YAP and TAZ knockdown RA-FLS. Reverse transcription quantitative real-time polymerase chain reaction (RT-qPCR) and western blotting were performed to screen the knockdown of YAP and TAZ.

### 2.3. Wound Healing Assay

RA-FLS were seeded into 6-well plates and cultured in serum-free medium overnight. Thereafter, a yellow tip was used to introduce a scratch in the confluent cell layer, and the cells were washed three times with phosphate-buffered saline (PBS) to remove the detached cells. Cells were cultured in an incubator of 5% CO_2_ at 37°C for 48 h, and cell scratch healings were photographed and measured using ImageJ software. The relative wound healing after 48 h is calculated. Each experiment was repeated three times.

### 2.4. Transwell Cell Migration and Invasion Assays

The Transwell assay was conducted as previously described [25]. Briefly, 2 × 10^4^ RA-FLS in 200 *μ*l serum-free DMEM per well were seeded into the uncoated or Matrigel- (100 *μ*g/ml, BD Biosciences) coated upper chamber for Transwell migration and invasion assays, respectively, and 600 *μ*l of the complete growth media (containing 12% FBS) was added to the lower chamber. After incubation at 37°C with 5% CO_2_ for 24 h, the migrated cells were fixed in methanol at room temperature for 15 min and stained with 0.1% crystal violet for 20 min. The cells remaining on the upper surface of the chamber membrane were gently removed with a swab. The chamber membranes were imaged at 100x or 200x magnification, and the cell counts of migration and invasion in five randomly selected microscopic fields under an inverted microscope were analyzed. All experiments were performed in triplicate from three independent experiments.

### 2.5. RT-qPCR

RT-qPCR was performed as previously described [25]. Briefly, TRIzol® reagent (Invitrogen, Carlsbad, CA, USA) was used to extract total RNA. Reverse transcription was performed by using the PrimeScript™ RT Master Mix (TaKaRa, Dalian, China). qPCR was conducted using a SYBR qPCR KIT (TaKaRa) on a LightCycler 96 System (Roche, Switzerland) according to the manufacturer's instructions, and the PCR thermocycling conditions were 95°C for 5 min, then 40 cycles of 95°C for 10 s followed by 60°C for 30 s. Gene-specific primers used in the present study were as follows: YAP forward, 5′-CTCGAACCCCAGATGACTTC-3′ and reverse, 5′-CCAGGAATGGCTTCAAGGTA-3′; TAZ forward, 5′-CTTGGATGTAGCCATGACCTT-3′ and reverse, 5′-TCAATCAAAACC AGGCAATG-3′; connective tissue growth factor (CTGF) forward, 5′-AGGAGT GGGTGTGTGACGA-3′ and reverse, 5′-CCAGGCAGTTGGCTCTAATC-3′; cysteine-rich angiogenic inducer 61 (CYR61) forward, 5′-AGCCTCGCATCCTATACAACC-3′ and reverse, 5′-TTCTTTCACAAGGCGGCACTC-3′; microtubule-associated protein 1 light chain 3 beta (LC3B) forward, 5′-AGCAGCATCCAACCAAAATC-3′ and reverse, 5′-TGTGTCCGTTCACCAACAG-3′; SQSTM1/p62 (p62) forward, 5′-ATCGGAGGATC CGAGTGT-3′ and reverse, 5′-TGGCTGTGAGCTGCTCTT-3′; unc-51-like autophagy activating kinase 1 (ULK1) forward, 5′-AAGATCGCTGACTTCGGCTT-3′ and reverse, 5′-TTCTCGTAGAACAGGCGCAG-3′; and glyceraldehyde-3-phosphate dehydrogenase (GAPDH) forward, 5′-GCACCGTCAAGGCTGAGAAC-3′ and reverse, 5′-TGGTGAAGA CGCCAGTGGA-3′. The relative levels of the mRNAs normalized against GAPDH mRNA were calculated by using the 2^-*ΔΔ*Ct^ methods. The experiment was independently repeated four times.

### 2.6. Western Blotting

The western blot procedure was carried out as previously described [25]. Briefly, RA-FLS were washed twice with cold PBS and lysed in RIPA lysis buffer containing 50 mM Tris, 0.15 M NaCl, 1 mM EGTA, 1% NP40, 0.25% SDS (Beyotime Institute of Biotechnology, Haimen, China), protease inhibitor mix, and phosphatase inhibitors (Roche Diagnostics, Switzerland). Samples were boiled and resolved with sodium dodecyl sulfate polyacrylamide gel electrophoresis (SDS-PAGE) and transferred onto polyvinylidene fluoride membranes (Millipore, Billerica, USA). Membranes were immunoblotted with primary antibodies followed by incubation with the horseradish peroxidase- (HRP-) conjugated secondary antibodies. The protein bands were visualized with the Pierce ECL Plus western blotting substrate (Thermo Fisher Scientific, Inc., USA), and GAPDH was used as the loading control. The following antibodies were used in the present study: rabbit anti-YAP (no. 14074), rabbit anti-TAZ (no. 4883), rabbit anti-ULK1 (no. 8054), rabbit anti-LC3B (no. 3868), rabbit anti-SQSTM1/p62 (no. 39749), rabbit anti-*β*-catenin (no. 8480), rabbit anti-Vimentin (no. 5741), HRP- linked anti-rabbit IgG (no. 7074), anti-mouse IgG (no. 7076) purchased from Cell Signaling Technology (Danvers, MA, USA), and mouse anti-GAPDH purchased from KangChen Bio-tech (Shanghai, China).

### 2.7. Immunofluorescence Analysis

RA-FLS were grown on sterile coverslips in 24-well plates and fixed with 4% paraformaldehyde in PBS for 30 min, permeabilized with 0.5% TritonX-100 for 15 min, blocked with Image-iT FX Signal Enhancer (Invitrogen), and then incubated with monoclonal rabbit anti-LC3B antibody at 4°C overnight. After washing three times with PBS, the cells were incubated with the rhodamine-conjugated goat anti-rabbit IgG (Biosource International) for 2 h at room temperature in the dark. Actin was visualized using rhodamine-conjugated phalloidin (Sigma-Aldrich, USA) for 2 h in the dark at room temperature and subsequently washed three times with PBS. Nuclei were counterstained with 0.5 *μ*g/ml DAPI (Sigma-Aldrich, USA). The fluorescence were captured using a fluorescence microscope at 1000x magnification.

### 2.8. Statistical Analysis

Data were presented as means ± standard deviation (SD). Statistical significance was determined by the Student *t*-test, and *P* values less than 0.05 was considered statistically significant.

## 3. Results

### 3.1. Upregulation of YAP and TAZ in RA-FLS

**Figure 1:**
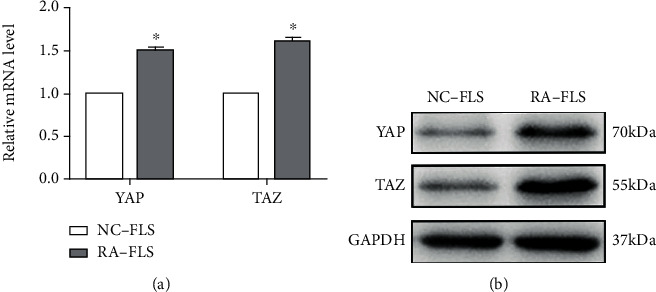
The expression of YAP and TAZ in normal FLS (NC-FLS) and RA-FLS. (a) YAP and TAZ mRNA levels were evaluated from NC-FLS and RA-FLS using RT-qPCR and normalized against GAPDH. Data are the mean ± SD (*n* = 4). ^∗^*P* < 0.05 relative to NC-FLS by Student's *t*-test. (b) Western blot analysis of TAZ and YAP proteins in NC-FLS and RA-FLS. GAPDH served as loading control. Representative blots are shown (*n* = 3).

**Figure 2:**
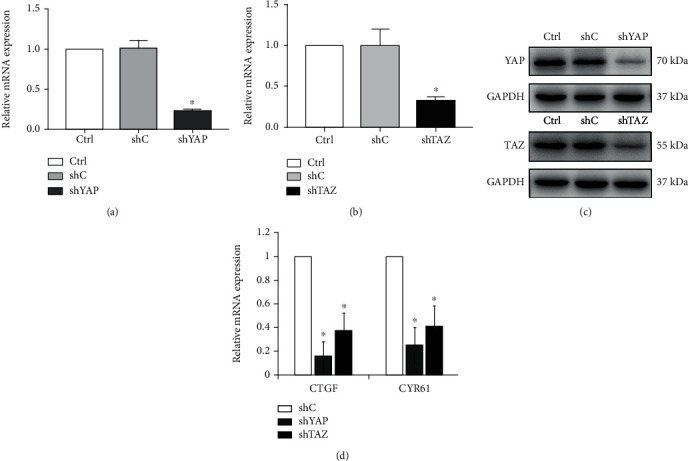
Knockdown of YAP and TAZ in the lentiviral-transduced RA-FLS. (a, b) YAP and TAZ mRNA levels were evaluated from RA-FLS stably expressing shRNA specific for YAP (shYAP), TAZ (shTAZ), the corresponding vector control (shC), and null (Ctrl) using RT-qPCR and normalized against GAPDH. Data are the mean ± SD (*n* = 4). ^∗^*P* < 0.05 relative to shC by Student's *t*-test. (c) Knockout of YAP and TAZ was confirmed by western blot analysis in RA-FLS, and GAPDH served as a loading control. Representative blots are shown (*n* = 3). (d) Quantitative analysis of CTGF and CYR61 mRNA in stable YAP and TAZ knockdown RA-FLS by RT-qPCR and normalized against GAPDH. Data were shown as the mean ± SD (*n* = 4). ^∗^*P* < 0.05 relative to shC by Student's *t*-test.

We first analyzed the expression of YAP and TAZ in normal FLS (NC-FLS) and RA-FLS by RT-qPCR and western blotting, respectively. We found that both mRNA and protein levels of YAP and TAZ in RA-FLS were obviously upregulated in RA-FLS compared with those in NC-FLS (see Figure 1), suggesting that YAP and TAZ might serve function as regulators in RA-FLS. Therefore, we assessed silencing of YAP and TAZ in RA-FLS by using the lentiviral-mediated YAP and TAZ knockdown techniques. Stable YAP and TAZ knockdown in RA-FLS was confirmed by RT-qPCR and western blotting (see Figures 2(a)–2(c)). Furthermore, we performed RT-qPCR to validate the effect of stable YAP or TAZ knockdown on the mRNA expression of CTGF and CYR61, which are well-known downstream targets of TAZ/YAP. The results showed that the mRNA levels of CTGF and CYR61 were significantly decreased in YAP or TAZ knocking down RA-FLS compared with the stably expressing control shRNA (shC) (see Figure 2(d)). Thus, RA-FLS with stable YAP or TAZ knockdown have been well established to perform the following experiments.

### 3.2. Knockdown of YAP or TAZ Inhibited Migration and Invasion of RA-FLS

**Figure 3:**
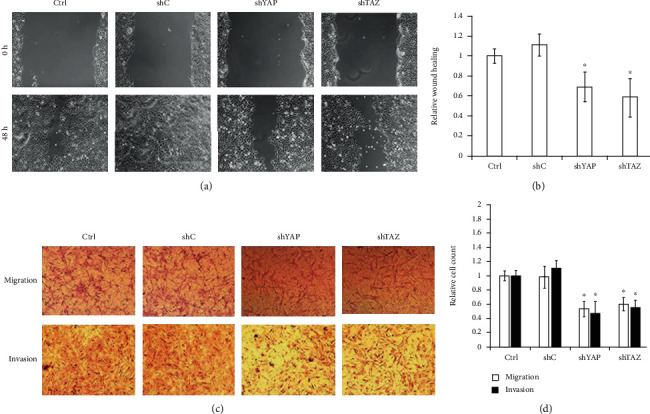
Knockdown of YAP or TAZ attenuated the migration and invasion of RA-FLS. (a, c) Migration and invasion of stable YAP or TAZ knockdown RA-FLS and their corresponding control cells (shC) and null (Ctrl) were determined by wound healing assays (a) and Transwell assays (c), respectively. Representative images of three independent experiments are shown. Magnification, ×100. (b, d) Quantification of relative numbers of migrated and invaded cells in five randomly fields for each replicate was shown. All experiments were performed independently three times, and data were shown as mean ± SD. ^∗^*P* < 0.05 relative to shC by Student's *t*-test.

**Figure 4:**
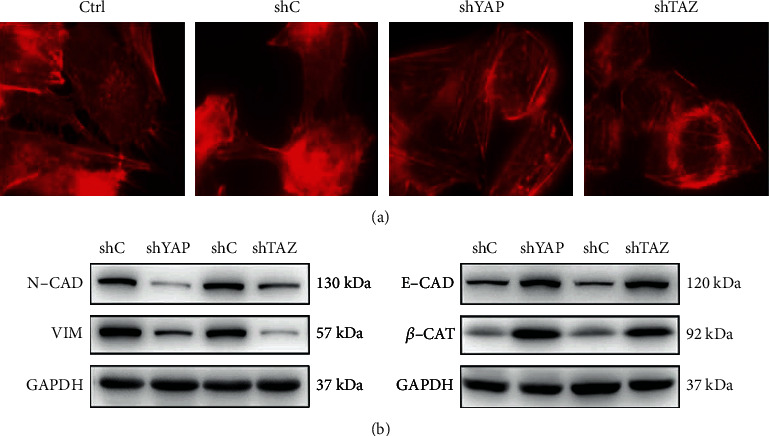
Knockdown of YAP or TAZ reduced epithelial-mesenchymal transition (EMT) of RA-FLS. (a) The distribution of actin fibers in stable YAP or TAZ knockdown RA-FLS was determined by immunofluorescence staining with rhodamine-labeled phalloidin, *n* = 3. Magnification, ×1000. (b) Protein levels of EMT markers were determined by western blot from RA-FLS stably expressing shRNA specific for YAP (shYAP), TAZ (shTAZ), and the corresponding vector control (shC), and GAPDH was used as a loading control. Representative blots are shown (*n* = 3).

It is well-known that YAP and TAZ can promote the migration and invasion of tumor cells, and RA-FLS have been likened to tumor-like cells due to their peculiar aggressive features [26]. Thus, we next performed wound healing assays and Transwell assays to evaluate whether YAP or TAZ knockdown affects the aggressive invasive ability of RA-FLS. As shown in Figure 3, RA-FLS with YAP or TAZ knockdown showed obvious reduction of wound closure in wound healing assays and significantly decreased the number of cells that migrated and invaded in Transwell assays compared with the stably expressing control shRNA cells, indicating that either YAP or TAZ knockdown can inhibit the migratory and invasive capacities of RA-FLS. To further confirm the effect of knockdown of YAP or TAZ on RA-FLS motility, we investigated the effect of YAP or TAZ knockdown on actin reorganization in RA-FLS by staining with phalloidin. RA-FLS with stable knockdown of YAP or TAZ displayed a thin or minimal lamellipodia and filopodia at the leading edge, which was responsible for actin reorganization to reduce cell locomotion (see Figure 4(a)). Since YAP and TAZ have been reported to contribute to multiple human cancers by regulating cell motility and EMT, we further evaluated the effect of knockdown of YAP or TAZ on the EMT-related markers in RA-FLS by western blotting assay. The results showed that either YAP or TAZ knockdown increased the expression of E-cadherin (E-CAD) and *β*-catenin (*β*-CAT) (epithelial marker) and decreased the expression of Vimentin (VIM) and N-cadherin (N-CAD) (mesenchymal marker) (see Figure 4(b)). These findings further explain the role of YAP/TAZ knockdown in RA-FLS motility, probably by modulation of the actin reorganization and the EMT process.

### 3.3. Knockdown of YAP or TAZ Promoted Autophagic Activity in RA-FLS

**Figure 5:**
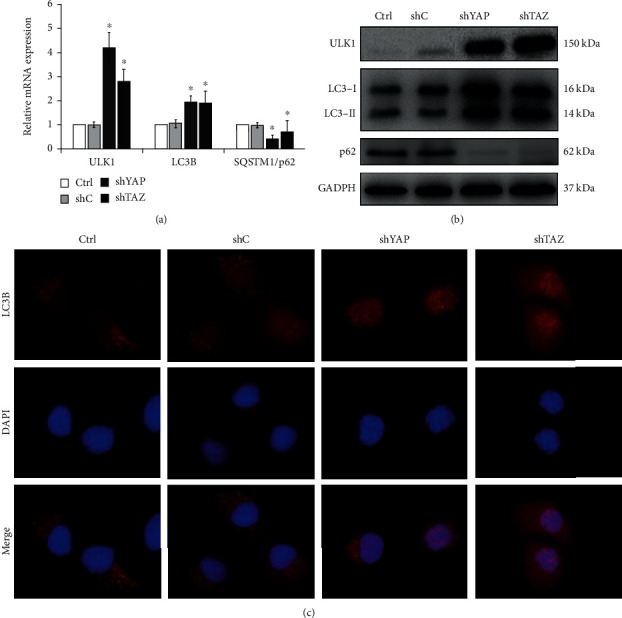
Knockdown of YAP or TAZ promoted autophagic activity in RA-FLS. (a) Quantitative analysis of mRNA levels of autophagy-related genes in stable YAP or TAZ knockdown RA-FLS by RT-qPCR and normalized against GAPDH. Data were shown as the mean ± SD (*n* = 4). ^∗^*P* < 0.05 relative to shC by Student's *t*-test. (b) Protein levels of ULK1, LC3B, and p62 were determined by western blot in stable YAP or TAZ knockdown RA-FLS, and GAPDH was used as a loading control. Representative blots are shown (*n* = 3). (c) LC3B puncta detected by immunofluorescence staining assay in stable YAP or TAZ knockdown RA-FLS (*n* = 3). Magnification, ×1000.

It has been reported that YAP-/TAZ-regulated autophagy is essential in normal cells and in tumor cells [27]; however, the effect of YAP/TAZ on autophagy in RA-FLS has not been elucidated. In order to define the relationship between YAP/TAZ and autophagy in RA-FLS, we first checked for the effect of knockdown of YAP/TAZ on the expression of autophagy-related genes by RT-qPCR and western blotting assays. Results from RT-qPCR showed that knockdown of YAP or TAZ significantly increased the expression of LC3B and ULK1, two markers of autophagosome formation and autophagy induction, and decreased the mRNA levels of SQSTM1/p62, a marker of autophagic degradation (see Figure 5(a)). Meanwhile, data from western blotting also demonstrated that loss of YAP or TAZ increased the levels of LC3B-II and ULK1 and reduced the levels of SQSTM1/p62 accumulation in RA-FLS (see Figure 5(b)). To further confirm these findings, we monitored autophagy through fluorescence microscopy in RA-FLS with stable knockdown of YAP or TAZ by following immunofluorescence staining of LC3B that represents autophagosome formation. YAP/TAZ knockdown increased the formation of LC3B spots in RA-FLS when compared with those in control-shRNA cells (see Figure 5(c)). The results demonstrated that knockdown of YAP/TAZ can promote autophagy activation in RA-FLS.

### 3.4. Knockdown of YAP or TAZ Inhibited Migration and Invasion of RA-FLS by Promoting Autophagy

**Figure 6:**
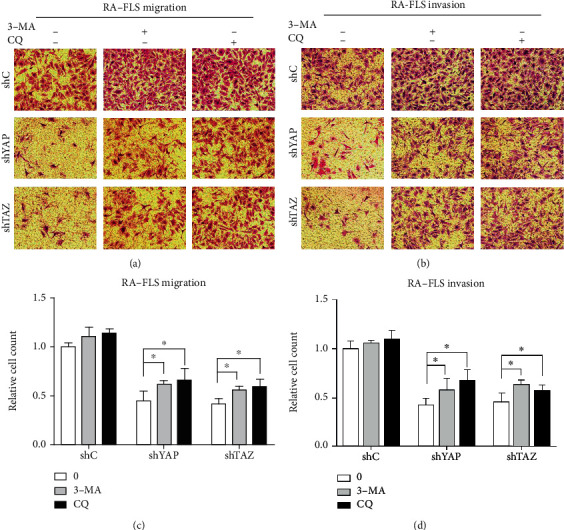
YAP or TAZ knockdown reduced the migratory and invasive ability of RA-FLS through autophagy induction. (a, b) Migration and invasion of stable YAP or TAZ knockdown RA-FLS and their corresponding control cells in the presence or absence of 10 mM 3-MA or 30 *μ*M CQ were determined by Transwell assays. Representative images of three independent experiments are shown. Magnification, ×200. (c, d) Quantification of relative numbers of migrated and invaded cells in five random fields for each replicate was shown. All experiments were performed independently three times, and data were shown as mean ± SD. ^∗^*P* < 0.05 relative to shYAP or shTAZ in the absence of 3-MA and CQ by Student's *t*-test.

**Figure 7:**
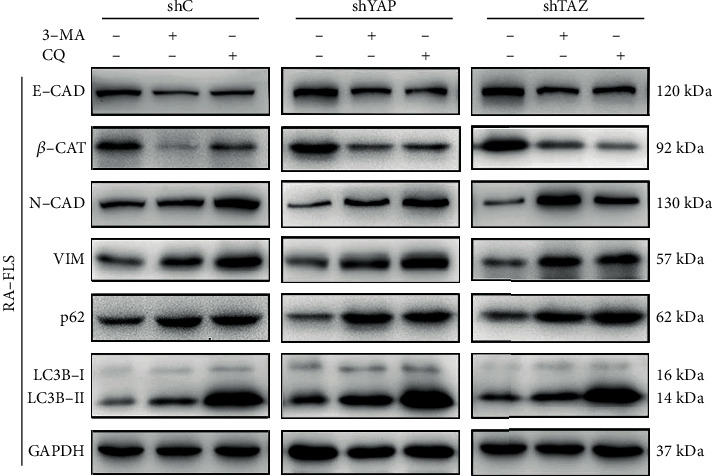
Effect of autophagy inhibition on the expression of the indicated genes in stable YAP or TAZ knockdown RA-FLS. The indicated proteins in cellular extracts were determined by western blot from RA-FLS in the presence or absence of 3-MA (10 mM) or CQ (30 *μ*M). GAPDH was used as a loading control. Representative blots are shown (*n* = 3).

Accumulating evidence indicates that autophagy plays a direct role in key aspects of tumor cell motility and invasion [28]. Since knockdown of YAP or TAZ can inhibit the migration and invasion and promote autophagy of RA-FLS as described above, we next evaluated whether the increased autophagic activity upon YAP or TAZ knockdown in RA-FLS reflects a decreased cell motility. To discriminate them, the effects of YAP or TAZ knockdown on migration and invasion of RA-FLS were studied in the presence of 3-methyladenine (3-MA), a class I phosphoinositide 3-kinase and class III phosphatidylinositol 3-kinase inhibitor used widely as a pharmacological inhibitor in autophagy studies, and chloroquine (CQ), an inhibitor of autophagosome degradation after the fusion of autophagosomes with lysosomes. Results from Transwell cell migration and invasion assays showed that treatment with 3-MA or CQ partially abrogated the migratory and invasive ability of RA-FLS induced by knockdown of YAP or TAZ (see Figure 6). To validate these findings, we next monitored the expression levels of LC3B, SQSTM1/p62, and EMT markers in RA-FLS with YAP or TAZ knockdown in the presence of 3-MA or CQ. The results showed that both 3-MA and CQ induced the accumulation of the amount of LC3B-II and significantly increased the amount of SQSTM1/p62 induced by either YAP or TAZ knockdown in RA-FLS (see Figure 7). In line with the results as mentioned above, significant reduction of the accumulation of E-cadherin and *β*-catenin and induction of N-cadherin and Vimentin in YAP- or TAZ-depleted RA-FLS after treatment with 3-MA or CQ were also observed (see Figure 7). Taken together, these findings support a role of YAP/TAZ knockdown inhibiting RA-FLS migration and invasion which may be attributed at least in part to the autophagy induction.

## 4. Discussion

The Hippo signaling pathway has emerged as a critical regulator of organ size control, tissue homeostasis, development, and tumor suppression by restricting cell proliferation and promoting apoptosis, and YAP and TAZ are downstream effectors of the Hippo signaling pathway [29, 30]; however, little is available yet on the role of the Hippo signaling in RA-FLS. Our former work demonstrated that both TAZ knockdown and knockout inhibited the migration, invasion, and EMT and promoted autophagy of the hepatocellular carcinoma cell lines [25]. Since RA-FLS present tumor-like characteristics of excessive migration and invasion towards cartilage and bone and play an important role in cartilage degradation and bone destruction [2–7], targeting the invasive property of RA-FLS is more important for RA therapy. Here, we report that RA-FLS display overexpression of YAP and TAZ. We provide evidence that silencing of YAP or TAZ inhibited the migration and invasion and promote autophagy of RA-FLS. Furthermore, we demonstrated the causal connection between cell motility and autophagy induced by silencing of YAP or TAZ.

In the current study, we clearly demonstrated that YAP and TAZ in RA-FLS exhibited higher expression than those in normal FLS, which was consistent with the results in multiple human cancer cells, implying that YAP and TAZ might serve function as the regulators in RA-FLS. Since RA-FLS can migrate and invade the articular cartilage and bone, thereby leading to destruction of cartilage and bone [6, 7], we firstly established RA-FLS stable knockdown of YAP or TAZ using the lentiviral-mediated YAP or TAZ knockdown approaches to evaluate the effect of YAP or TAZ knockdown on migration and invasion of RA-FLS by wound healing and Transwell migration and invasion assays. Our data demonstrated that either knockdown of YAP or TAZ markedly decreased cell motility, indicating the role of loss of YAP or TAZ resulting in the inhibition of the aggressive invasive ability of RA-FLS. Moreover, we also showed that silencing of YAP or TAZ altered rearrangement of the actin cytoskeleton. Since YAP/TAZ has been reported to involve in regulating EMT of cancer cells [31], we next detected the effect of YAP or TAZ knockdown on the EMT-related markers in RA-FLS. It was observed that either YAP or TAZ knockdown upregulated E-cadherin and *β*-catenin and downregulated N-cadherin and Vimentin. Although the exact molecular mechanism through which YAP or TAZ regulates the expression of EMT-related genes in RA-FLS remains to be clarified, we speculate that loss of YAP or TAZ contributes to the inhibition of cell motility through reduction of the EMT process in RA-FLS.

Recently, it has been demonstrated that autophagy plays an important role in the pathogenesis of some autoimmune diseases including RA [17, 18] so it is possible to provide new therapeutic targets for the treatment of the diseases by intervening or regulating the level of autophagy [32–34]. Although YAP and TAZ are involved in the regulation of autophagy in a variety of cells [27, 35–38], the effect of YAP/TAZ on the autophagy of RA-FLS is still unclear. The data presented in this manuscript has provided evidence for a role of YAP or TAZ knockdown in the positive regulation of autophagy of RA-FLS. This conclusion is supported by the observation that YAP or TAZ knockdown obviously increased the accumulation of LC3B-II and ULK1, two well-established markers of autophagosome formation and autophagy induction; decreased the amount of SQSTM1/p62, a marker indicating low autophagic activity; and enhanced the formation of LC3B spots in RA-FLS, which are distributed on the autophagosome membrane. Hence, our data delineate a role of loss of YAP/TAZ in regulating the autophagic degradation pathway in RA-FLS.

It has previously been demonstrated that autophagy can contribute to tumor cell migration and invasion[39, 40], and autophagy may serve function as either a tumor suppressor or a tumor promoter in tumor cell survival and motility [41, 42], suggesting that autophagy may play different roles in different cell types. Given that RA-FLS can migrate and invade the articular cartilage and bone, leading to destruction of cartilage and bone and joint damage, the contribution and relationship of knockdown of YAP or TAZ in the context of autophagy to RA-FLS migration and invasion remains to be clarified. Here, we demonstrated a role for autophagy activation induced by silencing of YAP or TAZ to inhibit migration and invasion of RA-FLS. Inhibition of autophagy either with 3-MA or CQ can partially reverse the decrease of migration and invasion caused by YAP/TAZ knockdown in RA-FLS. As is well-known, autophagy activation can suppress or strengthen EMT in cancer [43], and EMT is considered an important contributor in the process of cell migration and invasion. Our findings further confirmed that inhibition of autophagy by using 3-MA or CQ results in an increment of cell motility with the upregulation of N-cadherin and Vimentin and the reduction of the accumulation of E-cadherin and *β*-catenin in RA-FLS with stable knockdown of YAP or TAZ, suggesting that autophagy activation by YAP or TAZ knockdown acts to prevent EMT in RA-FLS. Taken together, these findings support a role of YAP/TAZ knockdown inhibiting RA-FLS migration and invasion which may be attributed, at least in part, to the autophagy induction. Thus, inhibiting the migration and invasion of RA-FLS by targeting autophagy might be a novel strategy for RA therapy.

In summary, the findings of the present study demonstrated that RA-FLS display overexpression of YAP and TAZ, and silencing of YAP or TAZ inhibited the migration and invasion and promote autophagy of RA-FLS. Our findings support a role of YAP/TAZ knockdown in inhibiting the migratory and invasive capacities of RA-FLS which might be attributed to the autophagy induction. Therefore, the causal connection between cell motility and autophagy induced by silencing of YAP or TAZ in RA-FLS might provide a promising target for the treatment of RA.

## 5. Conclusions

Our results indicated that silencing of YAP or TAZ inhibited the migration and invasion, promoted autophagy of RA-FLS, and displayed a role of YAP or TAZ knockdown in inhibiting the migratory and invasive capacities of RA-FLS which might be attributed to the autophagy induction. Therefore, the causal connection between cell motility and autophagy induced by silencing of YAP or TAZ in RA-FLS might provide a novel target for the treatment of RA.

## Data Availability

The data performed to support the findings of this study are included within the article.
